# Repressive Effect of Primary Virus Replication on Superinfection Correlated with Gut-Derived Central Memory CD4^+^ T Cells in SHIV-Infected Chinese Rhesus Macaques

**DOI:** 10.1371/journal.pone.0072295

**Published:** 2013-09-02

**Authors:** Jing Xue, Zhe Cong, Jing Xiong, Wei Wang, Hong Jiang, Ting Chen, Fangxin Wu, Kejian Liu, Aihua Su, Bin Ju, Zhiwei Chen, Marcelo A. Couto, Qiang Wei, Chuan Qin

**Affiliations:** 1 Institute of Laboratory Animal Science, Chinese Academy of Medical Sciences (CAMS) and Comparative Medicine Center, Peking Union Medical College (PUMC), Key Laboratory of Human Disease Comparative Medicine, Beijing, China; 2 AIDS Institute, Li Ka Shing Faculty of Medicine, The University of Hong Kong, Hong Kong SAR, China; 3 Department of Pathology and Laboratory Medicine, David Geffen School of Medicine at UCLA, Los Angeles, California, United States of America; University of Pittsburgh, United States of America

## Abstract

A possible mechanism of susceptibility to superinfection with simian-human immunodeficiency virus (SHIV)-1157ipd3N4 was explored in twelve SHIV_SF162P3_-infected Chinese rhesus macaques. Based on the kinetics of viral replication for the second infecting virus following SHIV-1157ipd3N4 inoculation, the monkeys were divided into two groups: those relatively resistant to superinfection (SIR) and those relatively sensitive to superinfection (SIS). We found that superinfection-resistant macaques had high primary viremia, whereas superinfection-sensitive macaques had low primary viremia, suggesting that primary SHIV_SF162P3_ infection with a high viral-replication level would repress superinfection with a heterologous SHIV-1157ipd3N4. Although no correlation of protection against superinfection with virus-specific CD4^+^ T cell or CD8^+^ T cell immune responses from gut was observed prior to superinfection, superinfection susceptibility was strongly correlated with CD4^+^ Tcm cells from gut both prior to the second infecting virus inoculation and on day 7 after superinfection, but not with CD4^+^ Tem cells from gut or with CD4^+^ Tcm cells from peripheral blood and lymph node. These results point to the important roles of gut-derived CD4^+^ Tcm cells for the study of the mechanisms of protection against superinfection and the evaluation of the safety and efficacy of vaccines and therapies against acquired immune deficiency syndrome (AIDS).

## Introduction

Superinfection with human immunodeficiency virus type 1 (HIV-1) is the infection of an HIV-seropositive individual with additional HIV-1 variants after a previous infecting strain has already become established. The first case of HIV-1 superinfection was reported in a chimpanzee model in 1987 [1] and in humans in 2000 [2]. Epidemiological studies have suggested that the frequency of superinfection ranges from rare to as high as 5% per year in high-risk populations [3]. The frequency of superinfection in different cohorts was recently summarized [4]. HIV-1 superinfection has become one of the main challenges in the prevention and treatment of acquired immune deficiency syndrome (AIDS).

The question of why superinfection occurs has not yet been completely answered. HIV-1 superinfection may depend on whether an HIV-specific immune response has been generated at the time of exposure to the second virus. A poorly protective immune response was originally suggested as the major factor responsible for superinfection. Superinfection by a second HIV-1 strain suggests that gaps in protective immunity might occur during natural infection. While previous studies on the association between the immune response to the primary HIV-1 virus and the superinfecting viruses covered antibodies [5]–[8], CD4^+^ T cells [9], [10] and CD8^+^ cytotoxic T cells [9], [11], they also produced conflicting results. Some suggested that the immune responses elicited by first HIV infection were sufficient to protect against superinfection, while others claimed the response was insufficient. In addition to the immune response, the characteristics of the viruses and the frequency of re-exposure might have also been involved in superinfection [12].

Since naturally occurring superinfection in humans is usually difficult to detect, this is undoubtedly an under-diagnosed phenomenon, which limits the opportunities for studying pathogenesis of superinfection in humans. Consequently, nonhuman primate models provide attractive platform for the study of superinfection, allowing deliberate viral exposures with known doses, strains, routes and timing of infection. While examining the extent of protection against superinfection conferred by the first infection and the biologic consequences of superinfection, Yeh et al. found that although the first SIV infection of rhesus macaques did not protect against subsequent mucosal challenge with a heterologous SIV isolate, the primary infection did attenuate the replication capacity of the second virus [13]. A correlation between susceptibility to superinfection and T cells from peripheral blood, however, was not observed in their study. In contrast, Salha et al. observed that the diversity of the CD4^+^ T-cell repertoire did play a role in SIV-infected macaques resistant to simian-human immunodeficiency virus (SHIV)89.6P superinfection [14]. Furthermore, Pahar et al. later demonstrated that primary infection of macaques with SHIV_SF162P3_ conferred partial to complete protection against subsequent challenge with the highly pathogenic SIVmac251 and suggested that the preservation of intestinal CD4^+^ memory T cells might be associated with protection from challenge [15].

**Figure 1:**
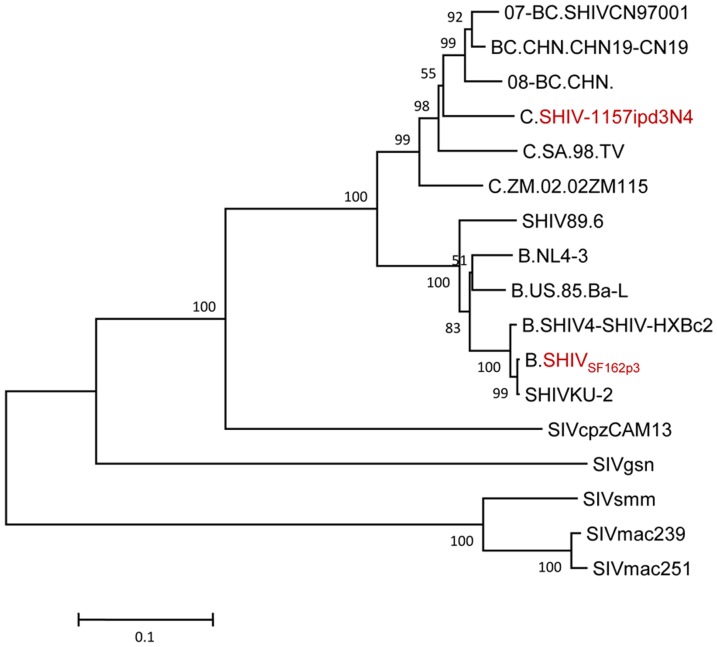
Phylogenetic analyses of the SHIV_SF162P3_ and SHIV-1157ipd3N4 env gene. The unrooted neighbor-joining phylogenetic tree from the full-length envelope gene was built using the MEGA5.1 software, and the numbers at each node represent bootstrap value (1000 replicates). Bootstrap values on branches are shown for the maximum neighbor-joining analyses. Reference samples were obtained from the Los Alamos National laboratory HIV database (http://hiv-web.lanl.gov/).

We chose to study lymphocytes derived from gut for two main reasons; 1) It is well known that the gastrointestinal tract is the major site of CD4^+^ T-cell depletion and viral replication in SIV/SHIV infection [16]–[18] and 2) Acute infection is usually accompanied by a marked depletion of CD4^+^ memory T cells, primarily from mucosal tissues [16], [19], [20]. Our choice of mucosally transmissible CCR5-tropic viruses for primary and secondary infections was based on the fact that gut-derived CD4^+^ T cells are the primary targets for CCR5-tropic SHIVs during primary infection [16], [21], [22], and previously published vaccine trials in rhesus macaques showed effective protection after CXCR4-tropic SHIV challenge but difficulty in containment of CCR5-tropic SIV/SHIV infection [23]–[25]. Moreover, in an analysis of infectious virus clones from two human cases of HIV-1 superinfection, van der Kuyl et al. found that superinfection occurred preferentially in patients infected with a relatively attenuated HIV-1 isolate. This suggested an effect of replication capacity of the primary HIV strain on superinfection [12]. In order to explore the effect of primary virus inoculation route and replication on superinfection, we enrolled in this study twelve SHIV_SF162P3_-infected Chinese rhesus macaques from a recently completed infection study. Six of these animals had high plasma viral levels (five from intravenous and one from intrarectal inoculation of the primary virus), and the other six animals had low plasma viral levels (all from intrarectal inoculation of the primary virus). To avoid the effect of replication capacity of primary virus on superinfection, SHIV-1157ipd3N4 was used for the second infection in this study. SHIV_SF162P3_ and SHIV-1157ipd3N4 are both pathogenic CCR5-tropic SHIVs. Although SHIV_SF162P3_ contains HIV clade B env gene and SHIV-1157ipd3N4 contains HIV clade C env gene (Fig. 1), their replication kinetics are quite similar [26]–[28]. Based on the viral replication kinetics of the second infecting virus, the monkeys were divided into two groups, i.e.: those that were relatively resistant to superinfection (SIR) and those that were relatively sensitive to superinfection (SIS). Here we observed a repressive effect on the replication of the superinfecting virus (SHIV-1157ipd3N4) by the primary infecting virus (SHIV_SF162P3_). Furthermore, we also found that superinfection susceptibility was positively correlated with gut-derived CD4^+^ Tcm cells both prior to and after superinfection, but not with gag-specific cellular immune responses by gut-derived CD4^+^ T cells or CD8^+^ T cells.

## Materials and Methods

### Ethics statement

The study protocol (No. ILAS-VL-2012-002) was approved by the Institutional Animal Care and Use Committee (IACUC) of the Institute of Laboratory Animal Science, Chinese Academy of Medical Sciences (ILAS, CAMS). All animal experimental procedures were performed in our Animal Bio-Safety Level 3 (ABSL-3) laboratory, which is fully accredited by the Association for Assessment and Accreditation of Laboratory Animal Care (AAALAC), International. This study was carried out in strict compliance with the “Guide for the Care and Use of Laboratory Animals of the Institute of Laboratory Animal Science (est. 2006)” and “The use of non-human primates in research of the Institute of Laboratory Animal Science (est. 2006)” to ensure the personnel safety and animal welfare.

All non-human primates used in this study were negative for SIV, SRV, STLV, B virus, TB, parasites (e.g., Entoameba), in accordance with national regulations (GB14922-2001). Monkeys were received into our quarantine facility upon arrival and underwent a full physical exam by a veterinarian approximately within 24 hours of arrival. Animals were anesthetized with ketamine hydrochloride for physical examination and weight determination and blood samples were obtained for CBC and chemistry panel.

Monkeys were housed in accordance with Chinese National standards, which are consistent with the standard set forth in the 8^th^ edition of the NRC Guide for the Care and Use of Laboratory Animals. Because of the infectious nature of this study, monkeys were housed individually instead of the generally recommended group or social housing. Stainless steel cages measuring 0.5–0.75 m^2^ and 0.5–0.6 m depending on the weight of the individual animal, consisted of wire flooring and resting boards or perches. Rooms have natural lighting and the photoperiod is supplemented during the winter months with an artificial lighting source to provide a 12∶12 light cycle. Temperature and humidity in animal holding rooms are maintained in accordance with recommendations in the Chinese National Standards for animal care. Drinking potable water is obtained from the city of Beijing and delivered to the animals via automated watering system (AWS). The AWS is checked daily to ensure proper operation i.e., water pressure, free flowing lixits and absence of leakages. Pans were cleaned daily and cages were washed every week by hand. All animals have individual cage ID cards which contain the following basic information: Study No., sex, weight, Principal Investigator's name and study protocol number. Monkeys were fed a measured amount of a commercially available monkey diet (Beijing HFK Bioscience Co., Ltd) offered twice daily. Fresh fruit (apples, bananas and oranges) are supplemented on alternating days.

Additional environmental enrichment consists of manipulanda, such as Kong toys, stainless steel mirrors and heavy-duty dog chew toys (Nyla bones or similar), which are provided on a rotating basis. Toys are left inside the cages when these are transported out of the room for washing and are sanitized at this time. Damaged toys are removed from circulation. Soft background music, plants, as well as pictures and photos hung on the animal room walls are provided for relaxation. Opportunities for limited social interaction with compatible monkeys are also provided at every other cage change when cages of compatible animals are placed in close proximity to each other while avoiding direct physical contact between animals.

Consistent with the practices followed in most other primate centers and facilities around the world, sedation with ketamine hydrochloride (10 mg/kg, i.m) is used whenever the animals must be handled. Because the experiments described herein involve inoculation with potentially lethal viruses, deep sedation is necessary to ensure the safety of the personnel during the direct manipulation of the animals.

We recognize that these experimental infections may cause varying degrees of discomfort, pain or distress to the animals. For this reason, we closely monitor all animals at least twice daily using the methodologies described previously [29]–[31]. The attending veterinarian is directly involved in all decisions regarding the clinical management of the animals. If the monkeys are deemed to be in pain or distress, appropriate drugs (anesthetics, analgesics or tranquillizers) are administered as prescribed by the veterinarian.

### Animals and viruses

The 12 Mamu-A*01-negative adult SHIV_SF162P3_-infected Chinese rhesus macaques used in this study were part of a recently completed infection study, and basic information for the twelve macaques is shown in Table 1. The two SHIVs used in this study were pathogenic CCR5-tropic SHIVs carrying distinct subtypes of the HIV-1 env gene as described elsewhere [26]–[28]. Briefly, SHIV_SF162P3_, with the HIV-1 clade B env gene, was obtained from Drs. Janet Harouse, Cecilia Cheng-Mayer, and Ranajit Pal through the NIAID (AIDS Research and Reference Reagent Program, NIH), and SHIV-1157ipd3N4, carrying the HIV-1 clade C env gene, was kindly provided by Dr. R. Ruprecht (obtained through the NIAID, NIH). A phylogenetic tree showed that the env genes in SHIV_SF162P3_ and SHIV-1157ipd3N4, are constructed with a common backbone from SIVmac239 and a chimerical env from a different HIV (Fig. 1). The two SHIVs are frequently used in nonhuman primate studies and were expanded on rhesus macaque peripheral blood mononuclear cells (PBMCs).

**Table 1:** Basic information for individual Chinese rhesus macaques with primary infections.

Monkey code	Sex	Weight (kg)	Primary infection virus	Route of primary infection^a^
G0802	♀	4.10	SHIV_SF162P3_	i.v.
G0803	♀	4.82	SHIV_SF162P3_	i.v.
G0804	♀	3.96	SHIV_SF162P3_	i.v.
G0805	♀	4.76	SHIV_SF162P3_	i.v.
G0806	♀	4.18	SHIV_SF162P3_	i.v.
G0812	♂	4.52	SHIV_SF162P3_	i.r.
G0813	♂	4.93	SHIV_SF162P3_	i.r.
G0814	♂	5.90	SHIV_SF162P3_	i.r.
G0815	♀	4.66	SHIV_SF162P3_	i.r.
G0817	♀	5.85	SHIV_SF162P3_	i.r.
G0819	♀	5.86	SHIV_SF162P3_	i.r.
G0821	♂	4.73	SHIV_SF162P3_	i.r.

a i.v., intravenous inoculation; i.r., intrarectal inoculation.

### Antibodies

The fluorescence-labeled monoclonal antibodies CD3 PerCP (SP34-2), IFN-γ PE-Cy7 (B27), IL-2 APC (MQ1-17H12) and mouse IgG1 and IgG2a isotype-matched controls were purchased from BD Biosciences (San Jose, CA). The monoclonal antibodies CD28 (CD28.2), CD49d (9F10), CD4 APC-Cy7 (OKT4), CD8 PE (RPA-T8), CD28 PE-Cy7 (CD28.2) and CD95 FITC (DX2) were obtained from Biolegend (San Diego, USA).

### qRT-PCR assay

Plasma viral loads were measured by a quantitative real-time reverse transcription-PCR (qRT-PCR) assay using a Perkin-Elmer ABI 7500 instrument, as described previously [31], [32]. The primers and probes were designed to anneal to regions of SHIV-1157ipd3N4-env and SHIV-gag that was shared by the two viral strains. The sequences for primers and probes are outlined in Table 2. Viral RNA was extracted and purified from cell-free plasma using the QIAmp viral RNA minikit (Qiagen, Valencia, CA). RNA was eluted in 20 µl of nuclease free water and frozen immediately at -80°C until analysis. SHIV_SF162P3_ RNA levels were determined using SHIV-gag primers and probes following primary infection, and SHIV-1157ipd3N4 RNA levels were specifically detected with SHIV-1157ipd3N4-env primers and probes following superinfection. The limits of detection for two viruses were 100 copy equivalents of RNA per ml of plasma. Triplicate test reactions were performed for each sample.

**Table 2:** Primers and probes for detection of viral loads by real-time PCR.

Gene	Primer	Sequence
SHIV-gag	Gag91F	5′-GCAGAGGAGGAAATTACCCAGTAC-3′
	Gag91R	5′-CAATTTTACCCAGGCATTTAATGTT-3′
	Probe	5′-FAM-ACCTGCCATTAAGCCCGA-MGB-3′
SHIV_1157ipd3N4_-env	115-4F	5′-CCATTATCGTTTCAGACCCACC-3′
	115-4R	5′-CCGGTCACTAATCGAATGGATC-3′
	Probe	5′-FAM-CCACTTCCGAGGGGAGCCGAC-TAMRA-3′

### Infection

Five SHIV_SF162P3_-infected monkeys were inoculated intravenously (SHIV_SF162P3_ at 40 TCID50, a single time) and seven were inoculated intrarectally (SHIV_SF162P3_ at 10 TCID50, several times until the detection of viral load). All animals were anesthetized with ketamine hydrochloride (10 mg/kg) prior to the procedures. SHIV-1157ipd3N4 was intravenously injected at 40 TCID50 on day 540 after primary infection to initiate superinfection. The experiments were performed in our Biosafety Level 3 Laboratory.

### Lymphocyte preparation

Peripheral blood was collected by venipuncture into EDTA tubes for the extraction and isolation of plasma by density gradient centrifugation. The inguinal lymph nodes of monkeys were isolated and biopsied at various time points. Gut biopsies were performed according to a standard protocol as previously described [33], [34]. After a 20-hr food fasting period, animals underwent intestinal biopsies using a 2.2-mm forceps (Fujinon Endoscop EG 250 WR5, Willich, Germany). Four to six biopsies were taken from the first 5 cm of the duodenum. Biopsies were immediately treated with 5 mM EDTA and 60 U/ml collagenase and isolated cells were enriched for lymphocytes by Percoll density gradient centrifugation.

### Flow cytometry analysis

Polychromatic flow cytometry was performed for staining of CD4^+^ T cell subsets. The surface-staining assay was performed as described previously [35], [36]. Briefly, collected cells were initially washed once with cold-flow wash buffer and stained for 1 hour at 4°C in the dark with the appropriately titrated antibodies (CD3 PerCP, CD4 APC-Cy7, CD8 PE, CD28 PE-Cy7 and CD95 FITC) for phenotyping of central memory (Tcm) and effector memory (Tem) CD4^+^ T cells, followed by two washes with cold-flow wash buffer. The cells were resuspended in 1% paraformaldehyde and subjected to flow cytometry analysis within 24 hours. Both compensation controls and fluorescence minus one (FMO) controls were utilized. Samples were acquired on a BD LSRII flow cytometer using FACSDiva Software (BD Biosciences) and FACS data were analyzed using FlowJo Version 8.7 (Tree Star, Ashland, USA). The percentages of CD4^+^ T, CD4^+^ Tcm and CD4^+^ Tem lymphocytes were calculated by multiplying the counts of CD3^+^ CD4^+^, CD28^+^ CD95^+^ and CD28^−^ CD95^+^ T lymphocytes by CD3^+^ T lymphocyte counts.

### Intracellular cytokine staining

Intracellular cytokine staining was performed as previously described [37]. Briefly, anti-CD28 and anti-CD49d monoclonal antibodies (as co-stimulatory molecules) were added to the lymphocytes purified from gut. Cells were then incubated at 37°C in a 5% CO_2_ environment for 8 hours in RPMI 1640 medium with 10% fetal bovine serum alone (mock stimulation), a pool of gag peptides (2 µg/ml, antigen-specific stimulation) or PMA (50 ng/ml, positive control), respectively. After surface staining with monoclonal antibodies (CD3 PerCP, CD4 APC-Cy7, CD8 PE), cells were fixed with Cytofix/Cytoperm solution (BD Biosciences), and then permeabilized and stained with antibodies (IFN-γ PE-Cy7, IL-2 APC) specific for IFN-γ and IL-2. Samples were acquired on a BD LSRII flow cytometer using FACSDiva Software, and FACS data were analyzed using FlowJo Version 8.7. Samples were considered positive when the percentage of cytokine-staining cells was three times that of the background.

### Statistical analysis

Data were represented as the mean ±SEM. Area under the curve (AUC) for the acute infection period and statistical analysis and graphical presentations were generated using GraphPad Prism scientific software. Comparisons between two groups were determined using the Mann-Whitney U test and the potential correlation between variables was analyzed with Pearson's correlation test. *P<0.05* was considered statistically significant.

## Results

### Repressive effect of primary virus replication on superinfecting virus replication

**Figure 2:**
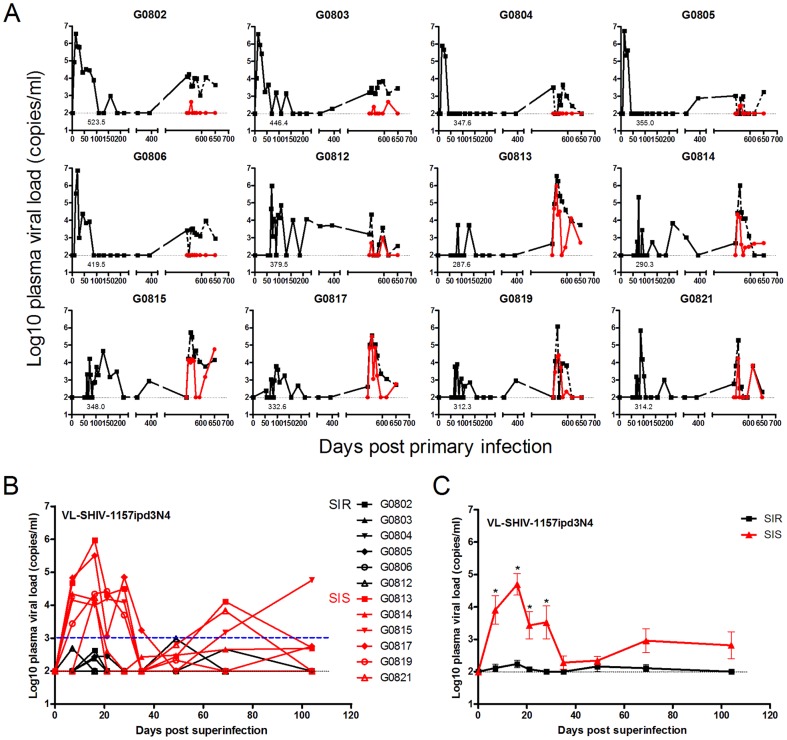
Plasma viral loads following primary infection and superinfection in each monkey. (**A**) Kinetics of plasma viral loads of twelve monkeys with a SHIV_SF162P3_ primary infection (black lines) followed by SHIV-1157ipd3N4 superinfection (red lines). Total viral loads of twelve monkeys after superinfection are shown by black dashed lines. The area under the curve (AUC) of SHIV_SF162P3_ viremia of each monkey for the acute infection (0–130 days) was indicated. (**B**) Kinetics of plasma SHIV-1157ipd3N4 loads in twelve monkeys after superinfection at each time point. The monkeys with plasma SHIV-1157ipd3N4 loads more than 10^3^ copies per ml (blue dashed line) were defined as superinfection-sensitive group (SIS) and those less than 10^3^ copies per ml as superinfection-resistant group (SIR). (**C**) Kinetics of the average viral load of SHIV-1157ipd3N4 in SIS (red line) and SIR monkeys (black line) (Mann-Whitney U test, *p<0.05). Viral loads are shown as log10 copies of viral load/ml of plasma and the limits of detection are 100 copies per ml (black dotted lines).

Five SHIV_SF162P3_-infected Chinese rhesus macaques inoculated intravenously (G0802, G0803, G0804, G0805 and G0806) and seven inoculated intrarectally (G0812, G0813, G0814, G0815, G0817, G0819 and G0821) were secondarily infected by intravenous inoculation of SHIV-1157ipd3N4 once the plasma SHIV_SF162P3_ RNA level had reached a plateau. Fig. 2A shows the replication kinetics of plasma SHIV_SF162P3_ and SHIV-1157ipd3N4 loads following the first and second infections. Peak SHIV_SF162P3_ viremia for G0802, G0803, G0804, G0805, G0806, G0812, G0814 and G0821 was approximately 10^5-7^ copies per ml and for G0813, G0815, G0817 and G0819 was as low as 10^3-5^ copies per ml. SHIV_SF162P3_ viremia was also represented by the area under the curve (AUC) for the acute infection (0-130 days). The AUC of SHIV_SF162P3_ viremia for G0802, G0803, G0805, G0806 and G0812 was approximately 350–550 and for G0804, G0813, G0814, G0815, G0817, G0819 and G0821 was approximately 280–350.

**Figure 3:**
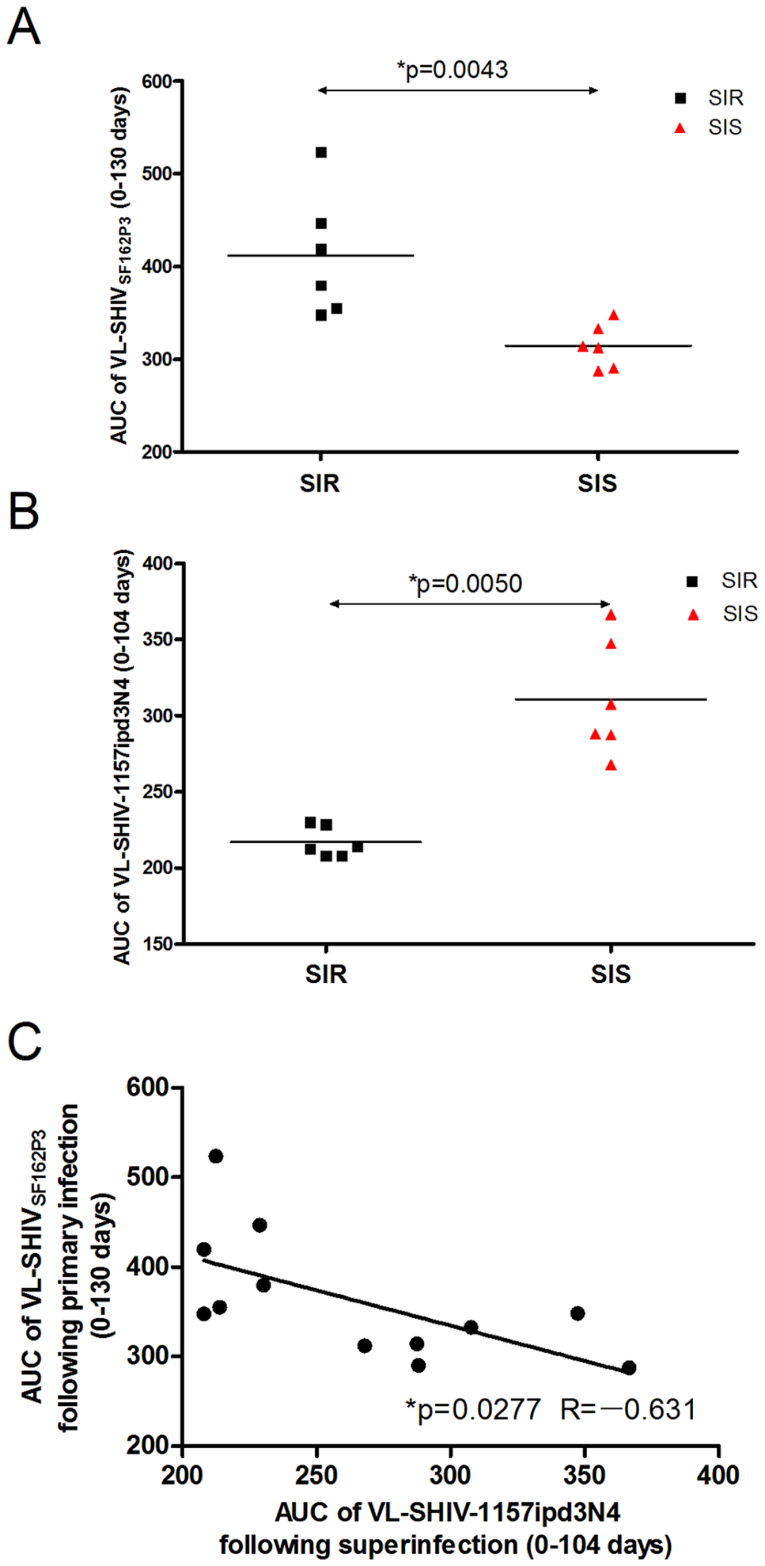
Repressive effect of SHIV_SF162P3_ replication on SHIV-1157ipd3N4 replication. (**A**) The AUC of SHIV_SF162P3_ viremia of SIS and SIR monkeys from day 0 to 130 after primary infection (Mann-Whitney U test, *P = 0.0043). (**B**) The AUC of SHIV-1157ipd3N4 viremia of SIS and SIR monkeys from day 0 to 104 after SHIV-1157ipd3N4 inoculation (Mann-Whitney U test, *P = 0.0050). (**C**) Correlation between the AUC of SHIV_SF162P3_ following primary infection and SHIV-1157ipd3N4 following superinfection (Pearson's correlation test, R = -0.631, *P = 0.0277).

After the second virus inoculation, twelve monkeys in this study manifested distinct outcomes and were divided into two groups (Fig. 2B) based on the kinetics of SHIV-1157ipd3N4 viral replication: 1) superinfection-resistant group (SIR), characterized by low plasma viral levels for SHIV-1157ipd3N4 RNA less than 10^3^ copies per ml for a very short time or transient (G0802, G0803, G0805 and G0812), and either no detection of plasma viral loads for SHIV-1157ipd3N4 RNA throughout the study (G0804 and G0806); 2) superinfection-sensitive group (SIS), defined as clear establishment of the second SHIV-1157ipd3N4 infection by high plasma viral levels for SHIV-1157ipd3N4 RNA more than 10^3^ copies per ml (G0813, G0814, G0815, G0817, G0819 and G0821). Plasma viral levels for SHIV-1157ipd3N4 RNA in SIS monkeys were maintained at 10^4-6^ copies per ml after the second virus inoculation and then declined gradually. Fig. 2C shows that mean plasma SHIV-1157ipd3N4 loads for SIS monkeys were significantly higher than those for SIR monkeys on days 7, 16, 21 and 28 (*P<0.05*). Fig. 3A shows that the AUC of SHIV_SF162P3_ after primary infection (0–130 days) were much higher for SIR monkeys than for SIS monkeys (*P = 0.0043*). In contrast, the AUC of SHIV-1157ipd3N4 after superinfection (0–104 days) were much lower for SIR monkeys than for SIS monkeys (*P = 0.0050*) (Fig. 3B). Fig. 3C shows a negative correlation between the AUC of SHIV_SF162P3_ and the AUC of SHIV-1157ipd3N4 (R = -0.631, *P = 0.0277*), suggesting a repressive effect of SHIV_SF162P3_ replication on SHIV-1157ipd3N4 replication. Interestingly, virtually all the macaques originally inoculated intravenously with SHIV_SF162P3_ were superinfection-resistant (SIR) and had high primary viremia; whereas 6/7 of the intrarectally inoculated macaques turned out to be superinfection-sensitive (SIS) and had low primary viremia.

### Correlation of superinfection with gut-derived CD4^+^ Tcm cells

**Figure 4:**
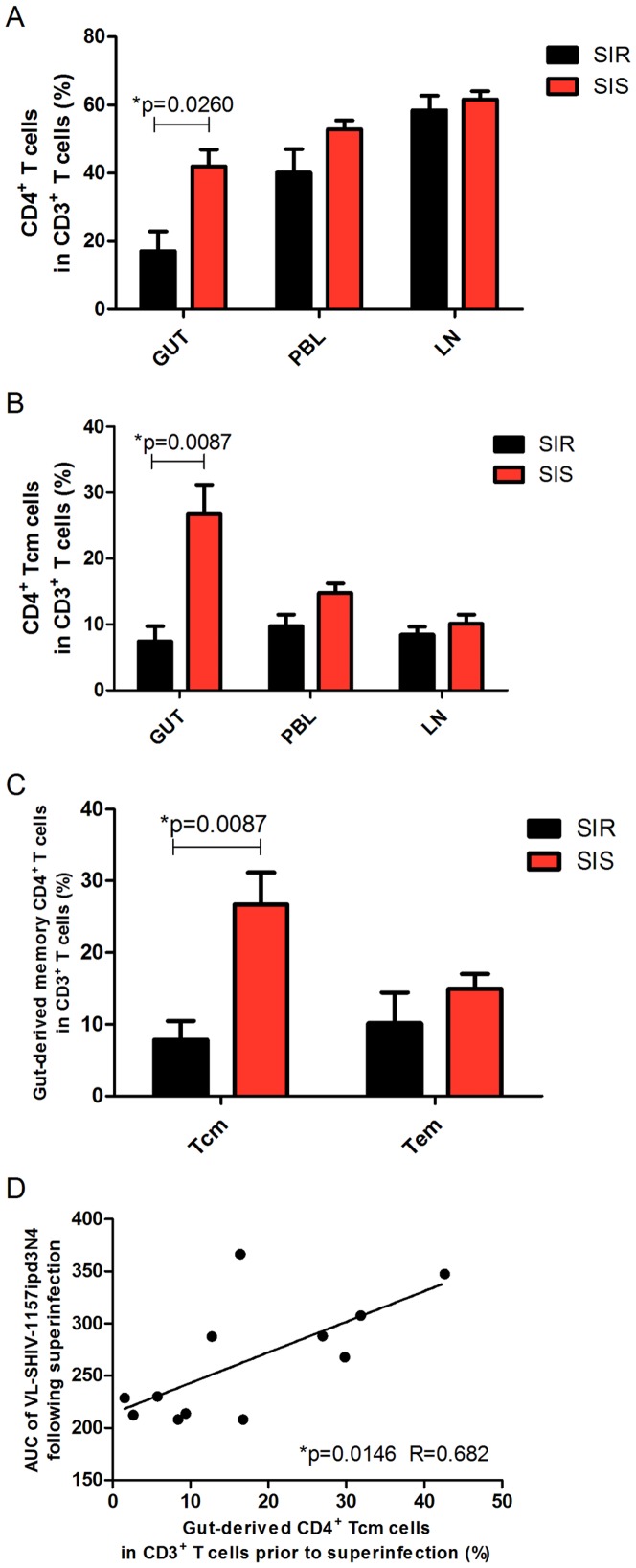
Correlation of superinfection with the percentage of CD4^+^ Tcm cells in CD3^+^ T cells from GUT prior to SHIV-1157ipd3N4 inoculation. (**A**) Percentages of CD4^+^ T cells in CD3^+^ T cells from PBL, LN and GUT in SIR monkeys (black bars) and SIS monkeys (red bars) (Mann-Whitney U test, *P = 0.0260). (**B**) Percentages of CD4^+^ Tcm cells in CD3^+^ T cells from PBL, LN and GUT in SIR monkeys (black bars) and SIS monkeys (red bars) (Mann-Whitney U test, *P = 0.0087). (**C**) Percentages of CD4^+^ Tcm cells or CD4^+^ Tem cells in CD3^+^ T cells from GUT in SIR (black bars) and SIS monkeys (red bars) (Mann-Whitney U test, *P = 0.0087). (**D**) Correlation between the AUC of SHIV-1157ipd3N4 viremia following superinfection and the percentage of gut-derived CD4^+^ Tcm cells in CD3^+^ T cells prior to SHIV-1157ipd3N4 inoculation (Pearson's correlation test, R = 0.682, *P = 0.0146). All the samples were obtained prior to SHIV-1157ipd3N4 inoculation (on day 540 after primary infection).

We examined the percentage of CD4^+^ T cells or CD4^+^ Tcm cells in CD3^+^ T lymphocytes from peripheral blood (PBL), lymph node (LN) and gut (GUT) prior to SHIV-1157ipd3N4 inoculation (on day 540 after primary infection) in the SIR and SIS groups. The data showed that the percentages of CD4^+^ T cells (Fig. 4A, *P* *= 0.0260*) or CD4^+^ Tcm cells (Fig. 4B, *P* *= 0.0087*) in CD3^+^ T lymphocytes from GUT but not PBL or LN were significantly lower for SIR monkeys than for SIS monkeys. However, no difference in the percentage of gut-derived CD4^+^ Tem cells in CD3^+^ T lymphocytes was observed between the two groups (Fig. 4C). In addition, it was found that the percentage of gut-derived CD4^+^ Tcm cells in CD3^+^ T lymphocytes prior to the second virus inoculation was positively correlated with the AUC of SHIV-1157ipd3N4 (R = 0.682, *P = 0.0146*) (Fig. 4D).

**Figure 5:**
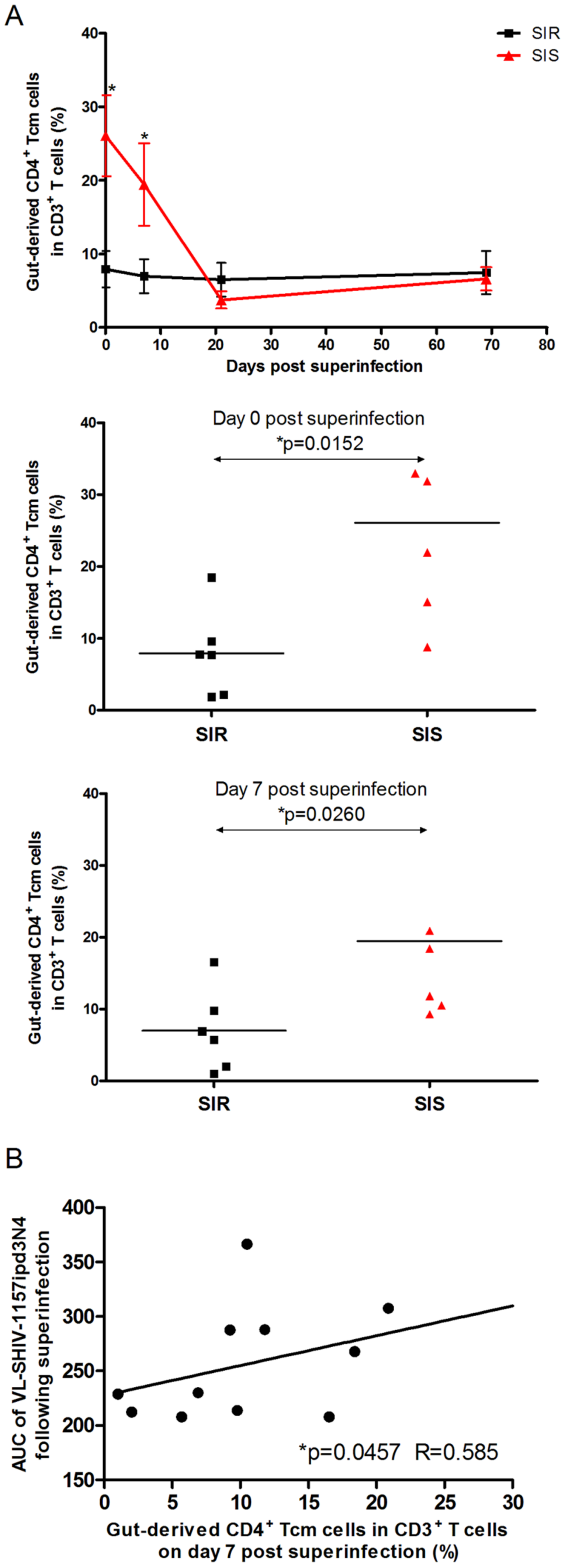
Correlation of superinfection with the percentage of CD4^+^ Tcm cells in CD3^+^ T cells from GUT after SHIV-1157ipd3N4 inoculation. (**A**) Percentage of gut-derived CD4^+^ Tcm cells in CD3^+^ T cells in SIS (red line) and SIR monkeys (black line) at each time point after superinfection, particularly on days 0 (*P = 0.0152) and 7 (*P = 0.0260) after SHIV-1157ipd3N4 inoculation (Mann-Whitney U test, *P<0.05). (**B**) Correlation between the AUC of SHIV-1157ipd3N4 viremia following superinfection and the percentage of gut-derived CD4^+^ Tcm cells in CD3^+^ T cells on day 7 after SHIV-1157ipd3N4 inoculation (Pearson's correlation test, R = 0.585, *P = 0.0457).

Fig. 5A shows the kinetics of the percentage of gut-derived CD4^+^ Tcm cells in CD3^+^ T lymphocytes after the second virus inoculation. For SIS monkeys, the percentage of gut-derived CD4^+^ Tcm cells in CD3^+^ T lymphocytes decreased gradually to the baseline after SHIV-1157ipd3N4 inoculation, while it kept on a low level in SIR monkeys during the whole study. At the time of SHIV-1157ipd3N4 inoculation (day 0), the percentage of gut-derived CD4^+^ Tcm cells in CD3^+^ T lymphocytes for SIS animals was greater than for SIR animals (*p = 0.0152*). On day 7 after SHIV-1157ipd3N4 inoculation, the difference of the percentage of gut-derived CD4^+^ Tcm cells in CD3^+^ T lymphocytes between the SIS and SIR monkeys was significant (*p = 0.0260*). Fig. 5B shows a positive correlation of the AUC of SHIV-1157ipd3N4 with the percentage of gut-derived CD4^+^ Tcm cells in CD3^+^ T lymphocytes on day 7 after SHIV-1157ipd3N4 inoculation (R = 0.585, *P = 0.0457*).

### No correlation of superinfection with SHIV gag-specific CD4^+^ or CD8^+^ T-cell responses from gut prior to SHIV-1157ipd3N4 inoculation

**Figure 6:**
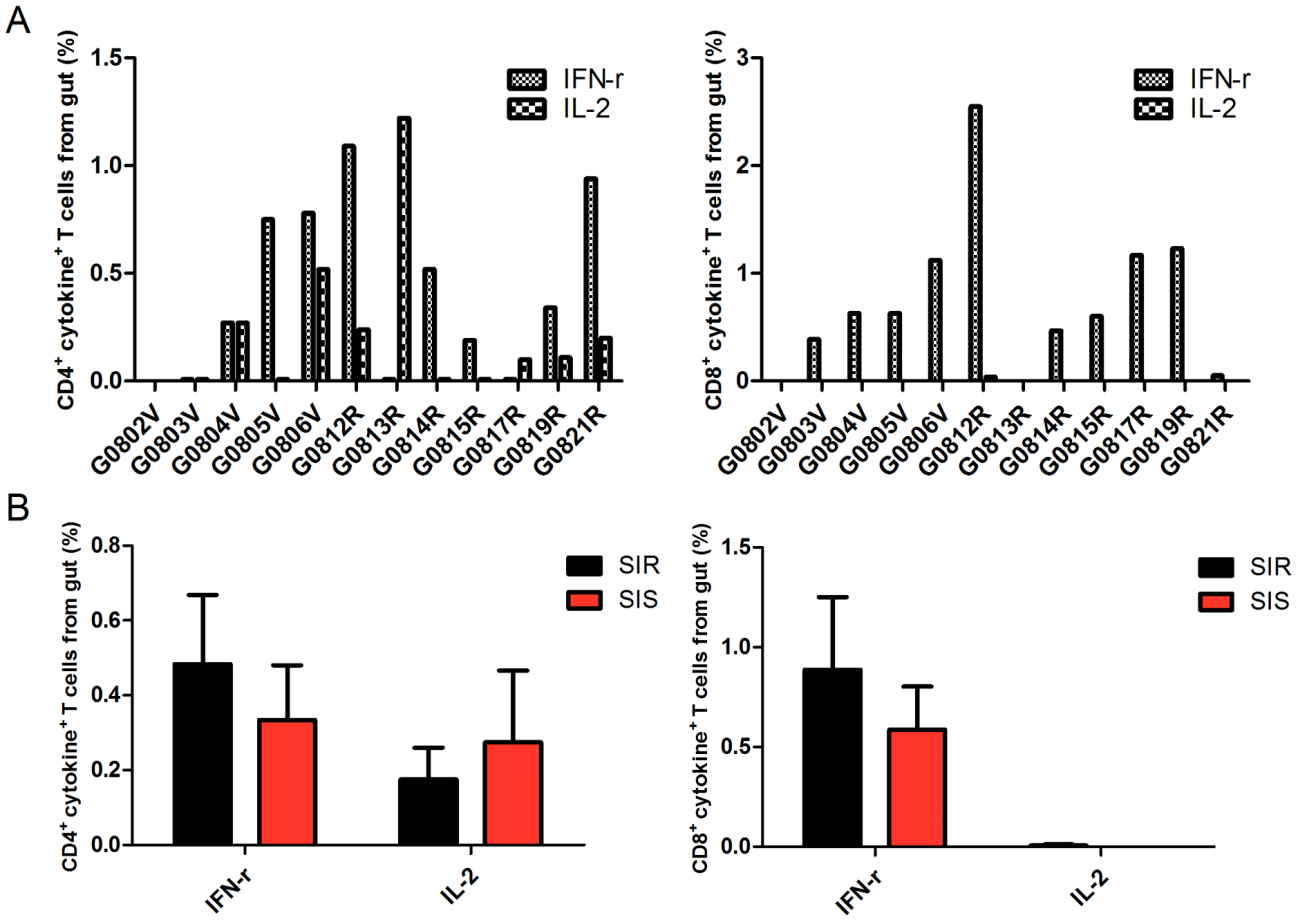
No correlation of superinfection with SHIV gag-specific CD4^+^ or CD8^+^ T-cell responses from GUT prior to SHIV-1157ipd3N4 inoculation.

To determine whether virus-specific cellular immune responses conferred protection against superinfection, all monkeys were evaluated for SHIV gag-specific T cellular immunity immediately prior to SHIV-1157ipd3N4 inoculation. Since CD4^+^ T cells from gut were reported to be the primary targets for CCR5-tropic viruses [16], [21], [22] and cytokine production was considered to be one of the major function of T cells in response to antigen stimulation, intracellular IFN-γ and IL-2 levels of gut-derived CD4^+^ T or CD8^+^ T cells from each monkey were assayed by flow cytometry after stimulation with SHIV gag peptides (Fig. 6A). PMA-induced intracellular IFN-γ and IL-2 production was assayed and 70–90% of CD4^+^ or CD8^+^ T cells from gut in SIS and SIR monkeys responded to the positive control PMA (data not shown). Fig. 6B shows that SHIV gag peptides-induced IFN-γ and IL-2 production by gut-derived CD4^+^ T cells or CD8^+^ T cells was indistinguishable between the SIS and SIR groups. These data suggest that SHIV gag-specific T cellular immune responses might not be responsible for the resistance of SHIV_SF162P3_-infected monkeys to SHIV-1157ipd3N4 superinfection.

## Discussion

HIV superinfection is one of the primary challenges to be overcome in the prevention and treatment of AIDS, yet the mechanism of superinfection remains poorly understood. It has been reported that the replication kinetics of SHIV_SF162P3_ and SHIV-1157ipd3N4 as well as the pathogenesis induced by SHIV_SF162P3_ or SHIV-1157ipd3N4 were quite similar to one another [26]–[28]. Therefore, we posit that the repressive effect of viral replication of the primary infection on superinfection observed in this study did not result from differences in the replication capability between the two viruses, but rather from the replication level of the first virus itself. Significant differences have been reported in the time of virus appearance, viral replication and magnitude of the immune responses between intravenous and intrarectal primary SIV/SHIV infection in macaques [38]–[40]. Our observations confirmed this. Yeh et al. found that the first SIV infection of rhesus macaques did not successfully protect against subsequent mucosal challenge with a heterologous SIV isolate. However, two macaques initially infected intravenously with SIVmac251 in their study were found to be resistant to superinfection with the heterologous virus SIVsmE660 [13]. Interestingly, we also found that virtually every superinfection-resistant macaque was originally inoculated intravenously with high primary viremia, whereas 6/7 of the rectally inoculated animals turned out to be susceptible to superinfection and had low primary viremia. It is known that the cellular receptors for HIV/SIV/SHIV entry include CD4 and several chemokine receptors such as CCR5. If CD4 and CCR5 receptors are occupied by the primary CCR5-tropic SHIV_SF162P3_ in some kind of saturation condition, they would likely block entry of the second CCR5-tropic SHIV-1157ipd3N4. Therefore, we can postulate that viral saturation of the cellular receptors or binding sites probably occurred in the superinfection-resistant monkeys as a direct result of higher viremia of the primary intravenous infection, which resulted in unavailability of the target cells for the superinfecting virus and were less permissive to replication of the superinfecting virus.

CD4^+^ T cells, especially the memory subsets of CD4^+^ T lymphocytes from gut, have long been considered to be the primary targets for CCR5-tropic viruses [16], [19], [20], [41], [42]. In this study, we found that the percentages of CD4^+^ T cells or CD4^+^ Tcm cells in CD3^+^ T cells from gut were greater in SIS monkeys than in SIR monkeys immediately prior to the second virus inoculation. This observation also supports the possibility that the target cells are less available for the superinfecting virus in SIR monkeys than in SIS monkeys.

CD4^+^ T cells in the intestinal mucosa are highly susceptible to SIV/HIV infection in both rhesus macaques [18] and humans [43]. Gut-associated lymphoid tissue (GALT) contains most of the body's lymphoid tissue and can be a persistent viral reservoir and an important site for host-pathogen interactions during HIV-1 infection [44], [45]. A correlation of SIV infection and disease progression with CD4^+^ T cells or CD4^+^ Tcm cells from gut has been reported. For example, Veazey et al. observed that SIV infection resulted in a profound and selective depletion of CD4^+^ T cells in the intestine within days of infection in rhesus monkeys [19]. Mason et al. reported that the depletion of CD28^+^ CD95^+^ central memory CD4^+^ T cells, but not other populations, was correlated with both SIV viral loads and disease progression [46]. Pahar et al. demonstrated that protection of the macaques against subsequent intravenous challenge with a highly pathogenic SIVmac251 by the primary infection with the minimally pathogenic virus SHIV_SF162P3_ might be associated with preservation of intestinal memory CD4^+^ CCR5^+^ T cells [15]. In our study, the percentage of CD4^+^ Tcm cells but not CD4^+^ Tem cells in CD3^+^ T cells from gut was found to be positively correlated with superinfection immediately prior to the second virus inoculation and on day 7 after superinfection. We also found that superinfection was not correlated with CD4^+^ T-cell counts (Fig. S1A and B), or with the percentage of CD4^+^ Tcm or CD4^+^ Tem cells in CD3^+^ T cells from peripheral blood prior to SHIV-1157ipd3N4 inoculation (Fig. S1C).

Live attenuated viruses as vaccines have provided more robust protection from severe heterologous viral challenge in non-human primate studies [47]–[50]. However, the debate is certain to continue regarding the potential mechanisms of the protection in terms of virus-specific immune responses. Some investigators were unable to identify a correlation between SIV-specific CD8 CTL responses elicited by inoculation with live attenuated SIV and protection against superinfection [51], [52]. Yeh et al. have suggested that there is no correlation of susceptibility to superinfection with virus-specific cellular immune responses in PBMCs of all monkeys. Our study also failed to uncover a correlation between superinfection resistance and SHIV gag-specific cellular immune responses by CD4^+^ or CD8^+^ T lymphocytes from the gut. This was consistent with the findings of other investigators [15]. However, Fukazawa et al. recently reported that live attenuated SIV vaccine-mediated protection against intravenous wildtype SIVmac239 challenge was strongly correlated with the magnitude and function of SIV-specific, effector-differentiated T cells in lymph node, but not with such T cell responses in peripheral blood or with other cellular, humoral and innate immune parameters [53]. Their study provides evidence that protection against a highly pathogenic strain of SIV may be achieved and associated with immune response in the germinal centers of lymph nodes. Although the difference of the percentage of CD4^+^ or CD4^+^ Tcm cells in CD3^+^ T cells from lymph nodes immediately prior to the second virus inoculation was not found in SIS and SIR monkeys in this study, it is unknown whether antigen specific responses of CD4^+^ or CD8^+^ T lymphocytes from lymph nodes confer protection against challenge with superinfecting SHIV-1157ipd3N4.

Efficient vaccine development is one of the most important projects for the prevention of HIV infection. However, the host immune responses to HIV vaccination have not always been found to be protective against HIV infection, and immune responses are not consistent with disease progression in many cases. Developing vaccines that can protect against infection by HIV virus is a daunting challenge. Superinfection offers an opportunity to examine the levels of anti-HIV immune response and to investigate the correlation between superinfection and anti-HIV immune responses. Nonhuman primate models have been widely used to evaluate the safety and efficacy of vaccines and medicines against AIDS [54]–[57]. These models will also provide important tools for the study of the mechanisms of susceptibility or resistance to superinfection. Investigations in nonhuman primates will likely provide important insights into vaccine development against AIDS.

## Supporting Information

Figure S1 No correlation of superinfection with CD4^+^ T-cell counts and the percentages of memory CD4^+^ T cells from peripheral blood (PBL) prior to SHIV-1157ipd3N4 inoculation. (**A**) Kinetics of CD4^+^ T-cell counts from PBL for twelve monkeys after primary infection and superinfection. (**B**) Correlation between the AUC of SHIV-1157ipd3N4 following superinfection and CD4^+^ T-cell counts from PBL prior to SHIV-1157ipd3N4 inoculation (Pearson's correlation test, R = 0.152, *P>0.05*). (**C**) No difference of the percentage of CD4^+^ Tcm or Tem cells in CD3^+^ T cells from PBL between SIS (red bars) and SIR monkeys (black bars) prior to SHIV-1157ipd3N4 inoculation (Mann-Whitney U test, *P>0.05*).
(DOC)
