# Supplementary figures and images for: Repressive Effect of Primary Virus Replication on Superinfection Correlated with Gut-Derived Central Memory CD4+ T Cells in SHIV-Infected Chinese Rhesus Macaques
Source: PLoS One. 2013 Sep 2;8(9):e72295. doi: 10.1371/journal.pone.0072295 (PMC3759369; doi:10.1371/journal.pone.0072295)

**
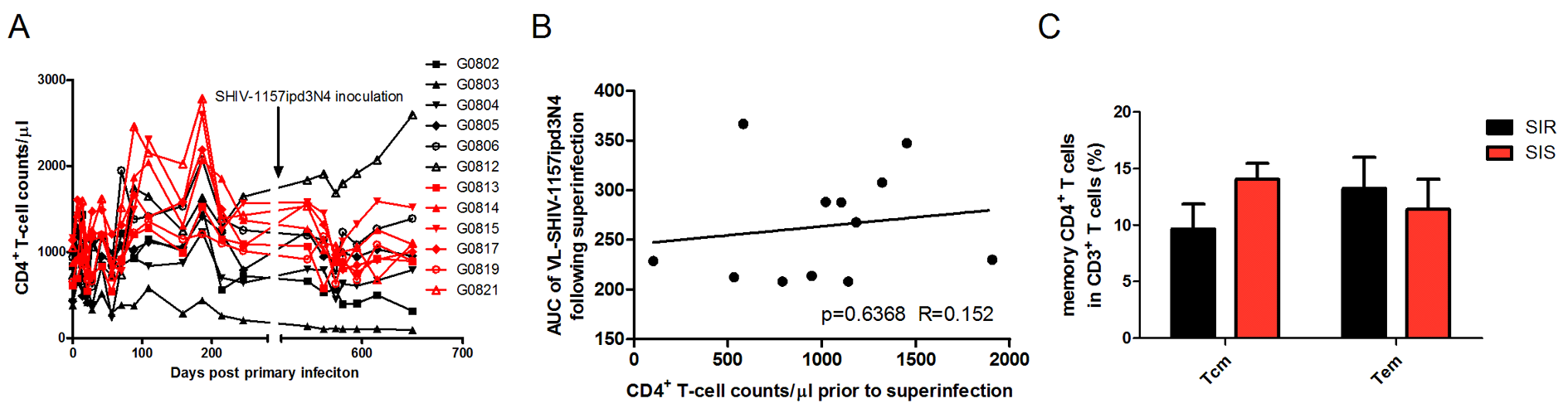
**

Supplement: Figure S1 — No correlation of superinfection with CD4+ T-cell counts and the percentages of memory CD4+ T cells from peripheral blood (PBL) prior to SHIV-1157ipd3N4 inoculation. (A) Kinetics of CD4+ T-cell counts from PBL for twelve monkeys after primary infection and superinfection. (B) Correlation between the AUC of SHIV-1157ipd3N4 following superinfection and CD4+ T-cell counts from PBL prior to SHIV-1157ipd3N4 inoculation (Pearson's correlation test, R = 0.152, P>0.05). (C) No difference of the percentage of CD4+ Tcm or Tem cells in CD3+ T cells from PBL between SIS (red bars) and SIR monkeys (black bars) prior to SHIV-1157ipd3N4 inoculation (Mann-Whitney U test, P>0.05). (DOC) [file pone.0072295.s001.doc]
